# Supplementary material for: Using Mendelian Randomisation to Prioritise Candidate Maternal Metabolic Traits Influencing Offspring Birthweight
Source: Metabolites. 2022 Jun 10;12(6):537. doi: 10.3390/metabo12060537 (PMC9231269; doi:10.3390/metabo12060537)
Supplement: Supplementary file 1 [file metabolites-12-00537-s001.zip › metabolites-1727089-supplementary.pdf]

Supplementary Table S1. UVMR effect estimates from UK Biobank selected SNPs (main analysis) and Kettunen selected SNPs.

Metabolite	Effect estimate (95% CI), UK Biobank selected SNPs	Pval., UK Biobank selected SNPs	Effect estimate (95% CI), Kettunen selected SNPs	Pval., Kettunen selected SNPs
3-Hydroxybutyrate	0.025 (-0.086, 0.137)	0.654	0.273 (0.144, 0.403)	<0.001
Acetate	0.107 (-0.147, 0.36)	0.409	--	--
Acetoacetate	0.008 (-0.142, 0.159)	0.915	0.052 (-0.186, 0.29)	0.666
Acetone	-0.083 (-0.207, 0.041)	0.188	--	--
Alanine	0.137 (0.036, 0.239)	0.008	0.011 (-0.111, 0.132)	0.862
Albumin	0.041 (-0.018, 0.101)	0.175	0.035 (-0.032, 0.101)	0.308
Apolipoprotein A1	-0.026 (-0.058, 0.006)	0.116	0.01 (-0.016, 0.037)	0.451
Apolipoprotein B	0.016 (-0.008, 0.041)	0.194	0.006 (-0.017, 0.03)	0.609
Citrate	-0.007 (-0.039, 0.024)	0.641	-0.029 (-0.079, 0.022)	0.264
Creatinine	-0.013 (-0.067, 0.041)	0.639	-0.02 (-0.084, 0.045)	0.549
Docosahexaenoic acid	0.004 (-0.025, 0.033)	0.796	0.046 (-0.013, 0.105)	0.124
Glucose	0.328 (0.219, 0.436)	0.000	0.259 (0.104, 0.414)	0.001
Glutamine	0.089 (0.033, 0.144)	0.002	0.071 (-0.049, 0.192)	0.247
Glycine	0.016 (-0.011, 0.042)	0.242	--	--
Glycoprotein acetyls	0.027 (-0.007, 0.06)	0.119	0 (-0.052, 0.053)	0.997
Histidine	0.058 (-0.08, 0.195)	0.412	0 (-0.085, 0.084)	0.995
Isoleucine	-0.046 (-0.17, 0.078)	0.469	-0.127 (-0.226, -0.029)	0.011
Lactate	-0.146 (-0.365, 0.073)	0.190	-0.022 (-0.386, 0.342)	0.906
Leucine	-0.012 (-0.156, 0.132)	0.871	-0.074 (-0.186, 0.038)	0.197
Linoleic acid	0.01 (-0.021, 0.04)	0.536	0.002 (-0.024, 0.028)	0.874
Monounsaturated fatty acids	0.003 (-0.024, 0.031)	0.806	0.013 (-0.049, 0.075)	0.687
Omega-3 fatty acids	0.001 (-0.021, 0.023)	0.939	0.006 (-0.067, 0.079)	0.873
Omega-6 fatty acids	0.007 (-0.021, 0.035)	0.642	0.006 (-0.027, 0.039)	0.718
Phenylalanine	-0.024 (-0.088, 0.04)	0.460	-0.055 (-0.115, 0.006)	0.076
Phosphatidylcholines	-0.001 (-0.034, 0.033)	0.973	-0.002 (-0.033, 0.029)	0.898
Pyruvate	-0.015 (-0.072, 0.041)	0.590	-0.135 (-0.239, -0.031)	0.011
Saturated fatty acids	0.002 (-0.032, 0.036)	0.904	0.023 (-0.04, 0.086)	0.470
Sphingomyelins	0.011 (-0.023, 0.045)	0.527	0.003 (-0.033, 0.038)	0.880
Total esterified cholesterol	0.008 (-0.021, 0.037)	0.592	--	--
Total free cholesterol	0.004 (-0.024, 0.033)	0.761	0.014 (-0.008, 0.037)	0.206
Total lipids in chylomicrons and extremely large VLDL	0.029 (-0.001, 0.059)	0.058	0.001 (-0.077, 0.08)	0.976
Total lipids in IDL	0.019 (-0.011, 0.048)	0.220	0.007 (-0.012, 0.027)	0.470
Total lipids in large HDL	-0.009 (-0.033, 0.016)	0.491	0.007 (-0.013, 0.027)	0.516
Total lipids in large LDL	0.011 (-0.014, 0.035)	0.396	0.01 (-0.009, 0.029)	0.307
Total lipids in large VLDL	0.026 (-0.003, 0.055)	0.083	0.005 (-0.049, 0.058)	0.864
Total lipids in medium HDL	-0.023 (-0.054, 0.008)	0.150	0.047 (-0.006, 0.1)	0.082
Total lipids in medium LDL	0.016 (-0.011, 0.043)	0.249	0.013 (-0.007, 0.033)	0.199
Total lipids in medium VLDL	0.017 (-0.012, 0.045)	0.253	0.008 (-0.039, 0.054)	0.748
Total lipids in small HDL	-0.013 (-0.054, 0.027)	0.526	0.001 (-0.036, 0.038)	0.951
Total lipids in small VLDL	0.013 (-0.014, 0.04)	0.337	0.002 (-0.037, 0.042)	0.907
Total lipids in very large HDL	0 (-0.026, 0.025)	0.978	-0.006 (-0.032, 0.02)	0.645
Total lipids in very small VLDL	0.007 (-0.016, 0.03)	0.544	0.006 (-0.021, 0.033)	0.645
Total phospholipids in lipoprotein particles	0.006 (-0.03, 0.041)	0.748	--	--

Total triglycerides	0.013 (-0.014, 0.041)	0.351	--	--
Tyrosine	0.031 (-0.037, 0.098)	0.371	0.002 (-0.042, 0.047)	0.911
Valine	-0.043 (-0.108, 0.023)	0.205	-0.012 (-0.061, 0.037)	0.642

CI: confidence interval; pval.: p-value. “--" indicating no SNPs were available for analyses.

Supplementary Table S2. The number of SNPs (K) representing each metabolite in UVMR analyses.

**(1) UKBB selected SNPs**		**(2) Kettunen selected SNPs**		**Number of overlapping SNPs (k)**
**Metabolite**	**SNPs (K)**	**Metabolite**	**SNPs (K)**	
3-Hydroxybutyrate	17	3-Hydroxybutyrate	1	0
Acetate	9	Acetate	0	0
Acetoacetate	7	Acetoacetate	2	1
Acetone	11	Acetone	0	0
Alanine	33	Alanine	6	2
Albumin	33	Albumin	4	0
Apolipoprotein A1	93	Apolipoprotein A1	13	1
Apolipoprotein B	68	Apolipoprotein B	25	3
Citrate	38	Citrate	5	0
Creatinine	69	Creatinine	6	1
Docosahexaenoic acid	61	Docosahexaenoic acid	6	0
Glucose	23	Glucose	4	1
Glutamine	47	Glutamine	5	2
Glycine	65	Glycine	0	0
Glycoprotein acetyls	71	Glycoprotein acetyls	9	0
Histidine	20	Histidine	5	1
Isoleucine	9	Isoleucine	1	1
Lactate	10	Lactate	2	0
Leucine	17	Leucine	3	1
Linoleic acid	63	Linoleic acid	17	2
Monounsaturated fatty acids	84	Monounsaturated fatty acids	7	2
Omega-3 fatty acids	75	Omega-3 fatty acids	6	2
Omega-6 fatty acids	67	Omega-6 fatty acids	12	2
Phenylalanine	14	Phenylalanine	4	0
Phosphatidylcholines	79	Phosphatidylcholine	8	2
Pyruvate	20	Pyruvate	1	1
Saturated fatty acids	69	Saturated fatty acids	7	2
Sphingomyelins	70	Sphingomyelins	8	1
Total esterified cholesterol	74	Total esterified cholesterol	0	0
Total free cholesterol	67	Total free cholesterol	19	1
Total lipids in chylomicrons and extremely large VLDL	83	Total lipids in chylomicrons and extremely large VLDL	7	2
Total lipids in IDL	69	Total lipids in IDL	28	2
Total lipids in large HDL	144	Total lipids in large HDL	18	2
Total lipids in large LDL	62	Total lipids in large LDL	31	2
Total lipids in large VLDL	90	Total lipids in large VLDL	9	3
Total lipids in medium HDL	83	Total lipids in medium HDL	6	1
Total lipids in medium LDL	56	Total lipids in medium LDL	26	1
Total lipids in medium VLDL	81	Total lipids in medium VLDL	11	3
Total lipids in small HDL	60	Total lipids in small HDL	12	1
Total lipids in small VLDL	97	Total lipids in small VLDL	15	3

Total lipids in very large HDL	123	Total lipids in very large HDL	17	2
Total lipids in very small VLDL	89	Total lipids in very small VLDL	23	2
Total phospholipids in lipoprotein particles	70	Total phospholipids in lipoprotein particles	0	0
Total triglycerides	90	Total triglycerides	12	2
Tyrosine	37	Tyrosine	6	1
Valine	24	Valine	5	0

Supplementary Table S3. Mean F-statistics calculated for UVMR models.

**(1) UKBB selected SNPs**		**(2) Kettunen selected SNPs**	
**Metabolite**	**F-statistic**	**Metabolite**	**F-statistic**
3-Hydroxybutyrate	64.084	3-Hydroxybutyrate	53.058
Acetate	58.695	Acetate	--
Acetoacetate	67.801	Acetoacetate	36.916
Acetone	61.027	Acetone	--
Alanine	71.857	Alanine	56.976
Albumin	80.183	Albumin	69.368
Apolipoprotein A1	107.162	Apolipoprotein A1	77.232
Apolipoprotein B	137.030	Apolipoprotein B	68.099
Citrate	121.023	Citrate	59.670
Creatinine	54.863	Creatinine	44.599
Docosahexaenoic acid	152.255	Docosahexaenoic acid	50.966
Glucose	68.441	Glucose	70.268
Glutamine	112.504	Glutamine	81.723
Glycine	296.942	Glycine	--
Glycoprotein acetyls	89.138	Glycoprotein acetyls	58.778
Histidine	75.065	Histidine	52.215
Isoleucine	96.513	Isoleucine	66.965
Lactate	59.865	Lactate	31.871
Leucine	82.782	Leucine	74.680
Linoleic acid	104.567	Linoleic acid	61.669
Monounsaturated fatty acids	106.689	Monounsaturated fatty acids	55.607
Omega-3 fatty acids	175.109	Omega-3 fatty acids	62.058
Omega-6 fatty acids	104.670	Omega-6 fatty acids	59.918
Phenylalanine	107.049	Phenylalanine	52.999
Phosphatidylcholines	120.368	Phosphatidylcholine	61.189
Pyruvate	98.857	Pyruvate	65.721
Saturated fatty acids	99.724	Saturated fatty acids	58.279
Sphingomyelins	132.187	Sphingomyelins	56.732
Total esterified cholesterol	115.212	Total esterified cholesterol	--
Total free cholesterol	119.797	Total free cholesterol	68.503
Total lipids in chylomicrons and extremely large VLDL	127.063	Total lipids in chylomicrons and extremely large VLDL	51.760
Total lipids in IDL	146.857	Total lipids in IDL	76.197
Total lipids in large HDL	115.069	Total lipids in large HDL	99.755
Total lipids in large LDL	150.708	Total lipids in large LDL	81.236
Total lipids in large VLDL	112.514	Total lipids in large VLDL	65.241
Total lipids in medium HDL	111.625	Total lipids in medium HDL	54.489
Total lipids in medium LDL	147.602	Total lipids in medium LDL	86.031
Total lipids in medium VLDL	105.435	Total lipids in medium VLDL	72.931

Total lipids in small HDL	96.463	Total lipids in small HDL	62.835
Total lipids in small VLDL	104.649	Total lipids in small VLDL	74.749
Total lipids in very large HDL	119.263	Total lipids in very large HDL	100.951
Total lipids in very small VLDL	135.738	Total lipids in very small VLDL	69.348
Total phospholipids in lipoprotein particles	114.359	Total phospholipids in lipoprotein particles	---
Total triglycerides	117.926	Total triglycerides	82.273
Tyrosine	100.275	Tyrosine	51.474
Valine	94.578	Valine	70.021

"---" is used where it was not possible to estimate the F-statistic for this metabolite.

Supplementary Table S4: MVMR effect estimates using UKBB selected SNPs.

Metabolite	Effect estimate (95% CI)	Pval.
Alanine	0.083 (-0.016, 0.181)	0.104
3-Hydroxybutyrate	-0.011 (-0.141, 0.119)	0.972
Glutamine	0.074 (0.019, 0.13)	0.011
Glucose	0.291 (0.193, 0.389)	<0.001
Isoleucine	-0.018 (-0.158, 0.123)	0.805
Pyruvate	-0.081 (-0.176, 0.013)	0.096

CI: confidence interval; pval.: p-value.

Supplementary Table S5. MVMR effect estimates using Kettunen selected SNPs .

Metabolite	Effect estimate (95% CI)	Pval.
Alanine	0.115 (-0.231, 0.462)	0.539
3-Hydroxybutyrate	0.322 (-0.33, 0.974)	0.370
Glutamine	0.076 (-0.106, 0.257)	0.445
Glucose	0.377 (0.093, 0.661)	0.041
Isoleucine	0.032 (-0.782, 0.846)	0.941
Pyruvate	-0.026 (-0.851, 0.8)	0.954

CI: confidence interval; ; pval.: p-value.

Supplementary Table S6. List of GWAS's included in SNP selection.

Analysis	Samples included in SNP selection	Independent sample from UKBB	Number of metabolic measures available	Sample size
UKBB GWAS	UKBB	No	46	115,078
Kettunen GWAS	Kettunen	Yes	42	24,925

Supplementary Table S7. UKBB NMR Metabolites included in analysis.

Biomarker name	Group	Subgroup	Included	Reason for exclusion
3-Hydroxybutyrate	Ketone bodies		Yes	--
Acetate	Ketone bodies		Yes	--
Acetoacetate	Ketone bodies		Yes	--
Acetone	Ketone bodies		Yes	--
Alanine	Amino acids		Yes	--
Albumin	Fluid balance		Yes	--
Apolipoprotein A1	Apolipoproteins		Yes	--
Apolipoprotein B	Apolipoproteins		Yes	--
Average diameter for HDL particles	Lipoprotein particle sizes		No	Representative of lipid composition within lipoprotein

Average diameter for LDL particles	Lipoprotein particle sizes		No	Representative of lipid composition within lipoprotein
Average diameter for VLDL particles	Lipoprotein particle sizes		No	Representative of lipid composition within lipoprotein
Cholesterol in chylomicrons and extremely large VLDL	Lipoprotein subclasses	Chylomicrons and extremely large VLDL (particle diameters from 75 nm upwards)	No	Representative of lipid composition within lipoprotein subclass
Cholesterol in IDL	Lipoprotein subclasses	IDL (average diameter 28.6 nm)	No	Representative of lipid composition within lipoprotein subclass
Cholesterol in large HDL	Lipoprotein subclasses	Large HDL (average diameter 12.1 nm)	No	Representative of lipid composition within lipoprotein subclass
Cholesterol in large LDL	Lipoprotein subclasses	Large LDL (average diameter 25.5 nm)	No	Representative of lipid composition within lipoprotein subclass
Cholesterol in large VLDL	Lipoprotein subclasses	Large VLDL (average diameter 53.6 nm)	No	Representative of lipid composition within lipoprotein subclass
Cholesterol in medium HDL	Lipoprotein subclasses	Medium HDL (average diameter 10.9 nm)	No	Representative of lipid composition within lipoprotein subclass
Cholesterol in medium LDL	Lipoprotein subclasses	Medium LDL (average diameter 23 nm)	No	Representative of lipid composition within lipoprotein subclass
Cholesterol in medium VLDL	Lipoprotein subclasses	Medium VLDL (average diameter 44.5 nm)	No	Representative of lipid composition within lipoprotein subclass
Cholesterol in small HDL	Lipoprotein subclasses	Small HDL (average diameter 8.7 nm)	No	Representative of lipid composition within lipoprotein subclass
Cholesterol in small LDL	Lipoprotein subclasses	Small LDL (average diameter 18.7 nm)	No	Representative of lipid composition within lipoprotein subclass
Cholesterol in small VLDL	Lipoprotein subclasses	Small VLDL (average diameter 36.8 nm)	No	Representative of lipid composition within lipoprotein subclass
Cholesterol in very large HDL	Lipoprotein subclasses	Very large HDL (average diameter 14.3 nm)	No	Representative of lipid composition within lipoprotein subclass
Cholesterol in very large VLDL	Lipoprotein subclasses	Very large VLDL (average diameter 64 nm)	No	Representative of lipid composition within lipoprotein subclass
Cholesterol in very small VLDL	Lipoprotein subclasses	Very small VLDL (average diameter 31.3 nm)	No	Representative of lipid composition within lipoprotein subclass
Cholesterol to total lipids ratio in chylomicrons and extremely large VLDL	Relative lipoprotein lipid concentrations	Chylomicrons and extremely large VLDL ratios	No	Derived measure
Cholesterol to total lipids ratio in IDL	Relative lipoprotein lipid concentrations	IDL ratios	No	Derived measure

Cholesterol to total lipids ratio in large HDL	Relative lipoprotein lipid concentrations	Large HDL ratios	No	Derived measure
Cholesterol to total lipids ratio in large LDL	Relative lipoprotein lipid concentrations	Large LDL ratios	No	Derived measure
Cholesterol to total lipids ratio in large VLDL	Relative lipoprotein lipid concentrations	Large VLDL ratios	No	Derived measure
Cholesterol to total lipids ratio in medium HDL	Relative lipoprotein lipid concentrations	Medium HDL ratios	No	Derived measure
Cholesterol to total lipids ratio in medium LDL	Relative lipoprotein lipid concentrations	Medium LDL ratios	No	Derived measure
Cholesterol to total lipids ratio in medium VLDL	Relative lipoprotein lipid concentrations	Medium VLDL ratios	No	Derived measure
Cholesterol to total lipids ratio in small HDL	Relative lipoprotein lipid concentrations	Small HDL ratios	No	Derived measure
Cholesterol to total lipids ratio in small LDL	Relative lipoprotein lipid concentrations	Small LDL ratios	No	Derived measure
Cholesterol to total lipids ratio in small VLDL	Relative lipoprotein lipid concentrations	Small VLDL ratios	No	Derived measure
Cholesterol to total lipids ratio in very large HDL	Relative lipoprotein lipid concentrations	Very large HDL ratios	No	Derived measure
Cholesterol to total lipids ratio in very large VLDL	Relative lipoprotein lipid concentrations	Very large VLDL ratios	No	Derived measure
Cholesterol to total lipids ratio in very small VLDL	Relative lipoprotein lipid concentrations	Very small VLDL ratios	No	Derived measure
Cholesteryl esters in chylomicrons and extremely large VLDL	Lipoprotein subclasses	Chylomicrons and extremely large VLDL (particle diameters from 75 nm upwards)	No	Representative of lipid composition within lipoprotein subclass
Cholesteryl esters in HDL	Cholesteryl esters		No	Representative of lipid composition within lipoprotein
Cholesteryl esters in IDL	Lipoprotein subclasses	IDL (average diameter 28.6 nm)	No	Representative of lipid composition within lipoprotein subclass
Cholesteryl esters in large HDL	Lipoprotein subclasses	Large HDL (average diameter 12.1 nm)	No	Representative of lipid composition within lipoprotein subclass
Cholesteryl esters in large LDL	Lipoprotein subclasses	Large LDL (average diameter 25.5 nm)	No	Representative of lipid composition within lipoprotein subclass
Cholesteryl esters in large VLDL	Lipoprotein subclasses	Large VLDL (average diameter 53.6 nm)	No	Representative of lipid composition within lipoprotein subclass
Cholesteryl esters in LDL	Cholesteryl esters		No	Representative of lipid composition within lipoprotein
Cholesteryl esters in medium HDL	Lipoprotein subclasses	Medium HDL (average diameter 10.9 nm)	No	Representative of lipid composition within lipoprotein subclass
Cholesteryl esters in medium LDL	Lipoprotein subclasses	Medium LDL (average diameter 23 nm)	No	Representative of lipid composition within lipoprotein subclass
Cholesteryl esters in medium VLDL	Lipoprotein subclasses	Medium VLDL (average diameter 44.5 nm)	No	Representative of lipid composition within lipoprotein subclass

Cholesteryl esters in small HDL	Lipoprotein sub-classes	Small HDL (average diameter 8.7 nm)	No	Representative of lipid composition within lipoprotein subclass
Cholesteryl esters in small LDL	Lipoprotein sub-classes	Small LDL (average diameter 18.7 nm)	No	Representative of lipid composition within lipoprotein subclass
Cholesteryl esters in small VLDL	Lipoprotein sub-classes	Small VLDL (average diameter 36.8 nm)	No	Representative of lipid composition within lipoprotein subclass
Cholesteryl esters in very large HDL	Lipoprotein sub-classes	Very large HDL (average diameter 14.3 nm)	No	Representative of lipid composition within lipoprotein subclass
Cholesteryl esters in very large VLDL	Lipoprotein sub-classes	Very large VLDL (average diameter 64 nm)	No	Representative of lipid composition within lipoprotein subclass
Cholesteryl esters in very small VLDL	Lipoprotein sub-classes	Very small VLDL (average diameter 31.3 nm)	No	Representative of lipid composition within lipoprotein subclass
Cholesteryl esters in VLDL	Cholesteryl esters		No	Representative of lipid composition within lipoprotein
Cholesteryl esters to total lipids ratio in chylomicrons and extremely large VLDL	Relative lipoprotein lipid concentrations	Chylomicrons and extremely large VLDL ratios	No	Derived measure
Cholesteryl esters to total lipids ratio in IDL	Relative lipoprotein lipid concentrations	IDL ratios	No	Derived measure
Cholesteryl esters to total lipids ratio in large HDL	Relative lipoprotein lipid concentrations	Large HDL ratios	No	Derived measure
Cholesteryl esters to total lipids ratio in large LDL	Relative lipoprotein lipid concentrations	Large LDL ratios	No	Derived measure
Cholesteryl esters to total lipids ratio in large VLDL	Relative lipoprotein lipid concentrations	Large VLDL ratios	No	Derived measure
Cholesteryl esters to total lipids ratio in medium HDL	Relative lipoprotein lipid concentrations	Medium HDL ratios	No	Derived measure
Cholesteryl esters to total lipids ratio in medium LDL	Relative lipoprotein lipid concentrations	Medium LDL ratios	No	Derived measure
Cholesteryl esters to total lipids ratio in medium VLDL	Relative lipoprotein lipid concentrations	Medium VLDL ratios	No	Derived measure
Cholesteryl esters to total lipids ratio in small HDL	Relative lipoprotein lipid concentrations	Small HDL ratios	No	Derived measure
Cholesteryl esters to total lipids ratio in small LDL	Relative lipoprotein lipid concentrations	Small LDL ratios	No	Derived measure
Cholesteryl esters to total lipids ratio in small VLDL	Relative lipoprotein lipid concentrations	Small VLDL ratios	No	Derived measure
Cholesteryl esters to total lipids ratio in very large HDL	Relative lipoprotein lipid concentrations	Very large HDL ratios	No	Derived measure

Cholesteryl esters to total lipids ratio in very large VLDL	Relative lipoprotein lipid concentrations	Very large VLDL ratios	No	Derived measure
Cholesteryl esters to total lipids ratio in very small VLDL	Relative lipoprotein lipid concentrations	Very small VLDL ratios	No	Derived measure
Citrate	Glycolysis related metabolites		Yes	--
Clinical LDL cholesterol	Cholesterol		No	Representative of lipid composition within lipoprotein
Concentration of chylomicrons and extremely large VLDL particles	Lipoprotein subclasses	Chylomicrons and extremely large VLDL (particle diameters from 75 nm upwards)	No	Representative of lipid composition within lipoprotein subclass
Concentration of HDL particles	Lipoprotein particle concentrations		No	Representative of lipid concentration within lipoprotein subclass
Concentration of IDL particles	Lipoprotein subclasses	IDL (average diameter 28.6 nm)	No	Representative of lipid concentration within lipoprotein subclass
Concentration of large HDL particles	Lipoprotein subclasses	Large HDL (average diameter 12.1 nm)	No	Representative of lipid concentration within lipoprotein subclass
Concentration of large LDL particles	Lipoprotein subclasses	Large LDL (average diameter 25.5 nm)	No	Representative of lipid composition within lipoprotein subclass
Concentration of large VLDL particles	Lipoprotein subclasses	Large VLDL (average diameter 53.6 nm)	No	Representative of lipid composition within lipoprotein subclass
Concentration of LDL particles	Lipoprotein particle concentrations		No	Representative of lipid concentration within lipoprotein subclass
Concentration of medium HDL particles	Lipoprotein subclasses	Medium HDL (average diameter 10.9 nm)	No	Representative of lipid composition within lipoprotein subclass
Concentration of medium LDL particles	Lipoprotein subclasses	Medium LDL (average diameter 23 nm)	No	Representative of lipid composition within lipoprotein subclass
Concentration of medium VLDL particles	Lipoprotein subclasses	Medium VLDL (average diameter 44.5 nm)	No	Representative of lipid composition within lipoprotein subclass
Concentration of small HDL particles	Lipoprotein subclasses	Small HDL (average diameter 8.7 nm)	No	Representative of lipid composition within lipoprotein subclass
Concentration of small LDL particles	Lipoprotein subclasses	Small LDL (average diameter 18.7 nm)	No	Representative of lipid composition within lipoprotein subclass
Concentration of small VLDL particles	Lipoprotein subclasses	Small VLDL (average diameter 36.8 nm)	No	Representative of lipid composition within lipoprotein subclass
Concentration of very large HDL particles	Lipoprotein subclasses	Very large HDL (average diameter 14.3 nm)	No	Representative of lipid composition within lipoprotein subclass

Concentration of very large VLDL particles	Lipoprotein subclasses	Very large VLDL (average diameter 64 nm)	No	Representative of lipid composition within lipoprotein subclass
Concentration of very small VLDL particles	Lipoprotein subclasses	Very small VLDL (average diameter 31.3 nm)	No	Representative of lipid composition within lipoprotein subclass
Concentration of VLDL particles	Lipoprotein particle concentrations		No	Representative of lipid concentration within lipoprotein subclass
Creatinine	Fluid balance		Yes	--
Degree of unsaturation	Fatty acids		No	Composite fatty acid measure
Docosahexaenoic acid	Fatty acids		Yes	--
Free cholesterol in chylomicrons and extremely large VLDL	Lipoprotein subclasses	Chylomicrons and extremely large VLDL (particle diameters from 75 nm upwards)	No	Representative of lipid composition within lipoprotein subclass
Free cholesterol in HDL	Free cholesterol		No	Representative of lipid composition within lipoprotein
Free cholesterol in IDL	Lipoprotein subclasses	IDL (average diameter 28.6 nm)	No	Representative of lipid composition within lipoprotein subclass
Free cholesterol in large HDL	Lipoprotein subclasses	Large HDL (average diameter 12.1 nm)	No	Representative of lipid composition within lipoprotein subclass
Free cholesterol in large LDL	Lipoprotein subclasses	Large LDL (average diameter 25.5 nm)	No	Representative of lipid composition within lipoprotein subclass
Free cholesterol in large VLDL	Lipoprotein subclasses	Large VLDL (average diameter 53.6 nm)	No	Representative of lipid composition within lipoprotein subclass
Free cholesterol in LDL	Free cholesterol		No	Representative of lipid composition within lipoprotein
Free cholesterol in medium HDL	Lipoprotein subclasses	Medium HDL (average diameter 10.9 nm)	No	Representative of lipid composition within lipoprotein subclass
Free cholesterol in medium LDL	Lipoprotein subclasses	Medium LDL (average diameter 23 nm)	No	Representative of lipid composition within lipoprotein subclass
Free cholesterol in medium VLDL	Lipoprotein subclasses	Medium VLDL (average diameter 44.5 nm)	No	Representative of lipid composition within lipoprotein subclass
Free cholesterol in small HDL	Lipoprotein subclasses	Small HDL (average diameter 8.7 nm)	No	Representative of lipid composition within lipoprotein subclass
Free cholesterol in small LDL	Lipoprotein subclasses	Small LDL (average diameter 18.7 nm)	No	Representative of lipid composition within lipoprotein subclass
Free cholesterol in small VLDL	Lipoprotein subclasses	Small VLDL (average diameter 36.8 nm)	No	Representative of lipid composition within lipoprotein subclass

Free cholesterol in very large HDL	Lipoprotein subclasses	Very large HDL (average diameter 14.3 nm)	No	Representative of lipid composition within lipoprotein subclass
Free cholesterol in very large VLDL	Lipoprotein subclasses	Very large VLDL (average diameter 64 nm)	No	Representative of lipid composition within lipoprotein subclass
Free cholesterol in very small VLDL	Lipoprotein subclasses	Very small VLDL (average diameter 31.3 nm)	No	Representative of lipid composition within lipoprotein subclass
Free cholesterol in VLDL	Free cholesterol		No	Representative of lipid composition within lipoprotein
Free cholesterol to total lipids ratio in chylomicrons and extremely large VLDL	Relative lipoprotein lipid concentrations	Chylomicrons and extremely large VLDL ratios	No	Derived measure
Free cholesterol to total lipids ratio in IDL	Relative lipoprotein lipid concentrations	IDL ratios	No	Derived measure
Free cholesterol to total lipids ratio in large HDL	Relative lipoprotein lipid concentrations	Large HDL ratios	No	Derived measure
Free cholesterol to total lipids ratio in large LDL	Relative lipoprotein lipid concentrations	Large LDL ratios	No	Derived measure
Free cholesterol to total lipids ratio in large VLDL	Relative lipoprotein lipid concentrations	Large VLDL ratios	No	Derived measure
Free cholesterol to total lipids ratio in medium HDL	Relative lipoprotein lipid concentrations	Medium HDL ratios	No	Derived measure
Free cholesterol to total lipids ratio in medium LDL	Relative lipoprotein lipid concentrations	Medium LDL ratios	No	Derived measure
Free cholesterol to total lipids ratio in medium VLDL	Relative lipoprotein lipid concentrations	Medium VLDL ratios	No	Derived measure
Free cholesterol to total lipids ratio in small HDL	Relative lipoprotein lipid concentrations	Small HDL ratios	No	Derived measure
Free cholesterol to total lipids ratio in small LDL	Relative lipoprotein lipid concentrations	Small LDL ratios	No	Derived measure
Free cholesterol to total lipids ratio in small VLDL	Relative lipoprotein lipid concentrations	Small VLDL ratios	No	Derived measure
Free cholesterol to total lipids ratio in very large HDL	Relative lipoprotein lipid concentrations	Very large HDL ratios	No	Derived measure
Free cholesterol to total lipids ratio in very large VLDL	Relative lipoprotein lipid concentrations	Very large VLDL ratios	No	Derived measure
Free cholesterol to total lipids ratio in very small VLDL	Relative lipoprotein lipid concentrations	Very small VLDL ratios	No	Derived measure
Glucose	Glycolysis related metabolites		Yes	--
Glutamine	Amino acids		Yes	--

Glycine	Amino acids		Yes	--
Glycoprotein acetyls	Inflammation		Yes	--
HDL cholesterol	Cholesterol		No	Representative of lipid composition within lipoprotein
Histidine	Amino acids		Yes	--
Isoleucine	Amino acids	Branched-chain amino acids	Yes	--
Lactate	Glycolysis related metabolites		Yes	--
LDL cholesterol	Cholesterol		No	Representative of lipid composition within lipoprotein
Leucine	Amino acids	Branched-chain amino acids	Yes	--
Linoleic acid	Fatty acids		Yes	--
Monounsaturated fatty acids	Fatty acids		Yes	--
Omega-3 fatty acids	Fatty acids		Yes	--
Omega-6 fatty acids	Fatty acids		Yes	--
Phenylalanine	Amino acids	Aromatic amino acids	Yes	--
Phosphatidylcholines	Other lipids		Yes	--
Phosphoglycerides	Other lipids		No	Representative of lipid composition within lipoprotein
Phospholipids in chylomicrons and extremely large VLDL	Lipoprotein subclasses	Chylomicrons and extremely large VLDL (particle diameters from 75 nm upwards)	No	Representative of lipid composition within lipoprotein subclass
Phospholipids in HDL	Phospholipids		No	Representative of lipid composition within lipoprotein subclass
Phospholipids in IDL	Lipoprotein subclasses	IDL (average diameter 28.6 nm)	No	Representative of lipid composition within lipoprotein subclasses
Phospholipids in large HDL	Lipoprotein subclasses	Large HDL (average diameter 12.1 nm)	No	Representative of lipid composition within lipoprotein subclasses
Phospholipids in large LDL	Lipoprotein subclasses	Large LDL (average diameter 25.5 nm)	No	Representative of lipid composition within lipoprotein subclasses
Phospholipids in large VLDL	Lipoprotein subclasses	Large VLDL (average diameter 53.6 nm)	No	Representative of lipid composition within lipoprotein subclasses
Phospholipids in LDL	Phospholipids		No	Representative of lipid composition within lipoprotein subclass
Phospholipids in medium HDL	Lipoprotein subclasses	Medium HDL (average diameter 10.9 nm)	No	Representative of lipid composition within lipoprotein subclasses
Phospholipids in medium LDL	Lipoprotein subclasses	Medium LDL (average diameter 23 nm)	No	Representative of lipid composition within lipoprotein subclasses
Phospholipids in medium VLDL	Lipoprotein subclasses	Medium VLDL (average diameter 44.5 nm)	No	Representative of lipid composition within lipoprotein subclasses

Phospholipids in small HDL	Lipoprotein subclasses	Small HDL (average diameter 8.7 nm)	No	Representative of lipid composition within lipoprotein subclasses
Phospholipids in small LDL	Lipoprotein subclasses	Small LDL (average diameter 18.7 nm)	No	Representative of lipid composition within lipoprotein subclasses
Phospholipids in small VLDL	Lipoprotein subclasses	Small VLDL (average diameter 36.8 nm)	No	Representative of lipid composition within lipoprotein subclasses
Phospholipids in very large HDL	Lipoprotein subclasses	Very large HDL (average diameter 14.3 nm)	No	Representative of lipid composition within lipoprotein subclasses
Phospholipids in very large VLDL	Lipoprotein subclasses	Very large VLDL (average diameter 64 nm)	No	Representative of lipid composition within lipoprotein subclasses
Phospholipids in very small VLDL	Lipoprotein subclasses	Very small VLDL (average diameter 31.3 nm)	No	Representative of lipid composition within lipoprotein subclasses
Phospholipids in VLDL	Phospholipids		No	Representative of lipid composition within lipoprotein subclass
Phospholipids to total lipids ratio in chylomicrons and extremely large VLDL	Relative lipoprotein lipid concentrations	Chylomicrons and extremely large VLDL ratios	No	Derived measure
Phospholipids to total lipids ratio in IDL	Relative lipoprotein lipid concentrations	IDL ratios	No	Derived measure
Phospholipids to total lipids ratio in large HDL	Relative lipoprotein lipid concentrations	Large HDL ratios	No	Derived measure
Phospholipids to total lipids ratio in large LDL	Relative lipoprotein lipid concentrations	Large LDL ratios	No	Derived measure
Phospholipids to total lipids ratio in large VLDL	Relative lipoprotein lipid concentrations	Large VLDL ratios	No	Derived measure
Phospholipids to total lipids ratio in medium HDL	Relative lipoprotein lipid concentrations	Medium HDL ratios	No	Derived measure
Phospholipids to total lipids ratio in medium LDL	Relative lipoprotein lipid concentrations	Medium LDL ratios	No	Derived measure
Phospholipids to total lipids ratio in medium VLDL	Relative lipoprotein lipid concentrations	Medium VLDL ratios	No	Derived measure
Phospholipids to total lipids ratio in small HDL	Relative lipoprotein lipid concentrations	Small HDL ratios	No	Derived measure
Phospholipids to total lipids ratio in small LDL	Relative lipoprotein lipid concentrations	Small LDL ratios	No	Derived measure
Phospholipids to total lipids ratio in small VLDL	Relative lipoprotein lipid concentrations	Small VLDL ratios	No	Derived measure
Phospholipids to total lipids ratio in very large HDL	Relative lipoprotein lipid concentrations	Very large HDL ratios	No	Derived measure

Phospholipids to total lipids ratio in very large VLDL	Relative lipoprotein lipid concentrations	Very large VLDL ratios	No	Derived measure
Phospholipids to total lipids ratio in very small VLDL	Relative lipoprotein lipid concentrations	Very small VLDL ratios	No	Derived measure
Polyunsaturated fatty acids	Fatty acids		No	Composite fatty acid measure
Pyruvate	Glycolysis related metabolites		Yes	--
Ratio of apolipoprotein B to apolipoprotein A1	Apolipoproteins		No	Derived measure
Ratio of docosahexaenoic acid to total fatty acids	Fatty acids	Fatty acid ratios	No	Derived measure
Ratio of linoleic acid to total fatty acids	Fatty acids	Fatty acid ratios	No	Derived measure
Ratio of monounsaturated fatty acids to total fatty acids	Fatty acids	Fatty acid ratios	No	Derived measure
Ratio of omega-3 fatty acids to total fatty acids	Fatty acids	Fatty acid ratios	No	Derived measure
Ratio of omega-6 fatty acids to omega-3 fatty acids	Fatty acids	Fatty acid ratios	No	Derived measure
Ratio of omega-6 fatty acids to total fatty acids	Fatty acids	Fatty acid ratios	No	Derived measure
Ratio of polyunsaturated fatty acids to monounsaturated fatty acids	Fatty acids	Fatty acid ratios	No	Derived measure
Ratio of polyunsaturated fatty acids to total fatty acids	Fatty acids	Fatty acid ratios	No	Derived measure
Ratio of saturated fatty acids to total fatty acids	Fatty acids	Fatty acid ratios	No	Derived measure
Ratio of triglycerides to phosphoglycerides	Other lipids		No	Derived measure
Remnant cholesterol (non-HDL, non-LDL -cholesterol)	Cholesterol		No	Derived measure
Saturated fatty acids	Fatty acids		Yes	----
Sphingomyelins	Other lipids		Yes	
Total cholesterol	Cholesterol		No	
Total cholesterol minus HDL-C	Cholesterol		No	Derived measure
Total cholines	Other lipids		No	
Total concentration of branched-chain amino acids (leucine + isoleucine + valine)	Amino acids	Branched-chain amino acids	No	Derived measure
Total concentration of lipoprotein particles	Lipoprotein particle concentrations		No	

Total esterified cholesterol	Cholesteryl esters		Yes	--
Total fatty acids	Fatty acids		No	Composite fatty acid measure
Total free cholesterol	Free cholesterol		Yes	--
Total lipids in chylomicrons and extremely large VLDL	Lipoprotein subclasses	Chylomicrons and extremely large VLDL (particle diameters from 75 nm upwards)	Yes	--
Total lipids in HDL	Total lipids		No	
Total lipids in IDL	Lipoprotein subclasses	IDL (average diameter 28.6 nm)	Yes	--
Total lipids in large HDL	Lipoprotein subclasses	Large HDL (average diameter 12.1 nm)	Yes	--
Total lipids in large LDL	Lipoprotein subclasses	Large LDL (average diameter 25.5 nm)	Yes	--
Total lipids in large VLDL	Lipoprotein subclasses	Large VLDL (average diameter 53.6 nm)	Yes	--
Total lipids in LDL	Total lipids		No	
Total lipids in lipoprotein particles	Total lipids		No	
Total lipids in medium HDL	Lipoprotein subclasses	Medium HDL (average diameter 10.9 nm)	Yes	--
Total lipids in medium LDL	Lipoprotein subclasses	Medium LDL (average diameter 23 nm)	Yes	--
Total lipids in medium VLDL	Lipoprotein subclasses	Medium VLDL (average diameter 44.5 nm)	Yes	--
Total lipids in small HDL	Lipoprotein subclasses	Small HDL (average diameter 8.7 nm)	Yes	--
Total lipids in small LDL	Lipoprotein subclasses	Small LDL (average diameter 18.7 nm)	No	High correlation
Total lipids in small VLDL	Lipoprotein subclasses	Small VLDL (average diameter 36.8 nm)	Yes	--
Total lipids in very large HDL	Lipoprotein subclasses	Very large HDL (average diameter 14.3 nm)	Yes	--
Total lipids in very large VLDL	Lipoprotein subclasses	Very large VLDL (average diameter 64 nm)	No	High correlation
Total lipids in very small VLDL	Lipoprotein subclasses	Very small VLDL (average diameter 31.3 nm)	Yes	--
Total lipids in VLDL	Total lipids		No	
Total phospholipids in lipoprotein particles	Phospholipids		Yes	--
Total triglycerides	Triglycerides		Yes	--
Triglycerides in chylomicrons and extremely large VLDL	Lipoprotein subclasses	Chylomicrons and extremely large VLDL (particle diameters from 75 nm upwards)	No	
Triglycerides in HDL	Triglycerides		No	
Triglycerides in IDL	Lipoprotein subclasses	IDL (average diameter 28.6 nm)	No	
Triglycerides in large HDL	Lipoprotein subclasses	Large HDL (average diameter 12.1 nm)	No	

Triglycerides in large LDL	Lipoprotein subclasses	Large LDL (average diameter 25.5 nm)	No	
Triglycerides in large VLDL	Lipoprotein subclasses	Large VLDL (average diameter 53.6 nm)	No	
Triglycerides in LDL	Triglycerides		No	
Triglycerides in medium HDL	Lipoprotein subclasses	Medium HDL (average diameter 10.9 nm)	No	
Triglycerides in medium LDL	Lipoprotein subclasses	Medium LDL (average diameter 23 nm)	No	
Triglycerides in medium VLDL	Lipoprotein subclasses	Medium VLDL (average diameter 44.5 nm)	No	
Triglycerides in small HDL	Lipoprotein subclasses	Small HDL (average diameter 8.7 nm)	No	
Triglycerides in small LDL	Lipoprotein subclasses	Small LDL (average diameter 18.7 nm)	No	
Triglycerides in small VLDL	Lipoprotein subclasses	Small VLDL (average diameter 36.8 nm)	No	
Triglycerides in very large HDL	Lipoprotein subclasses	Very large HDL (average diameter 14.3 nm)	No	
Triglycerides in very large VLDL	Lipoprotein subclasses	Very large VLDL (average diameter 64 nm)	No	
Triglycerides in very small VLDL	Lipoprotein subclasses	Very small VLDL (average diameter 31.3 nm)	No	
Triglycerides in VLDL	Triglycerides		No	
Triglycerides to total lipids ratio in chylomicrons and extremely large VLDL	Relative lipoprotein lipid concentrations	Chylomicrons and extremely large VLDL ratios	No	Derived measure
Triglycerides to total lipids ratio in IDL	Relative lipoprotein lipid concentrations	IDL ratios	No	Derived measure
Triglycerides to total lipids ratio in large HDL	Relative lipoprotein lipid concentrations	Large HDL ratios	No	Derived measure
Triglycerides to total lipids ratio in large LDL	Relative lipoprotein lipid concentrations	Large LDL ratios	No	Derived measure
Triglycerides to total lipids ratio in large VLDL	Relative lipoprotein lipid concentrations	Large VLDL ratios	No	Derived measure
Triglycerides to total lipids ratio in medium HDL	Relative lipoprotein lipid concentrations	Medium HDL ratios	No	Derived measure
Triglycerides to total lipids ratio in medium LDL	Relative lipoprotein lipid concentrations	Medium LDL ratios	No	Derived measure
Triglycerides to total lipids ratio in medium VLDL	Relative lipoprotein lipid concentrations	Medium VLDL ratios	No	Derived measure
Triglycerides to total lipids ratio in small HDL	Relative lipoprotein lipid concentrations	Small HDL ratios	No	Derived measure
Triglycerides to total lipids ratio in small LDL	Relative lipoprotein lipid concentrations	Small LDL ratios	No	Derived measure
Triglycerides to total lipids ratio in small VLDL	Relative lipoprotein lipid concentrations	Small VLDL ratios	No	Derived measure

Triglycerides to total lipids ratio in very large HDL	Relative lipoprotein lipid concentrations	Very large HDL ratios	No	Derived measure
Triglycerides to total lipids ratio in very large VLDL	Relative lipoprotein lipid concentrations	Very large VLDL ratios	No	Derived measure
Triglycerides to total lipids ratio in very small VLDL	Relative lipoprotein lipid concentrations	Very small VLDL ratios	No	Derived measure
Tyrosine	Amino acids	Aromatic amino acids	Yes	--
Valine	Amino acids	Branched-chain amino acids	Yes	--
VLDL cholesterol	Cholesterol		No	Representative of lipid composition within lipoprotein subclass

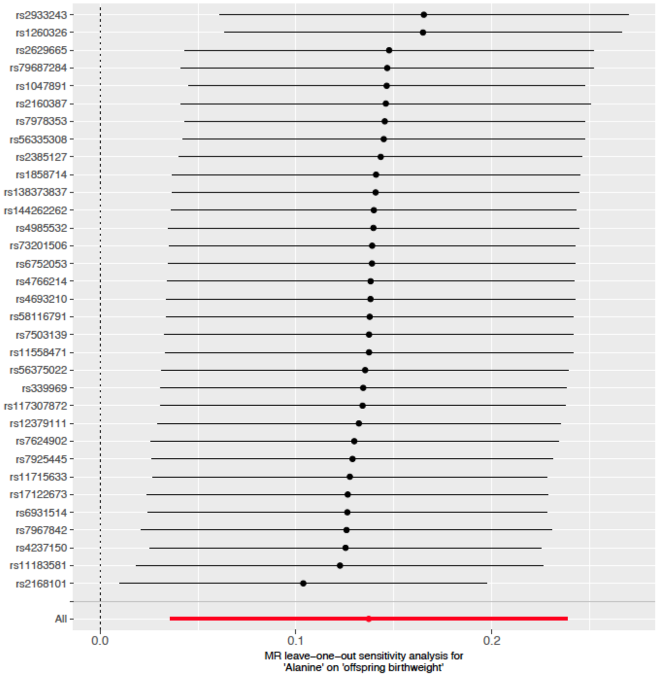

(A)

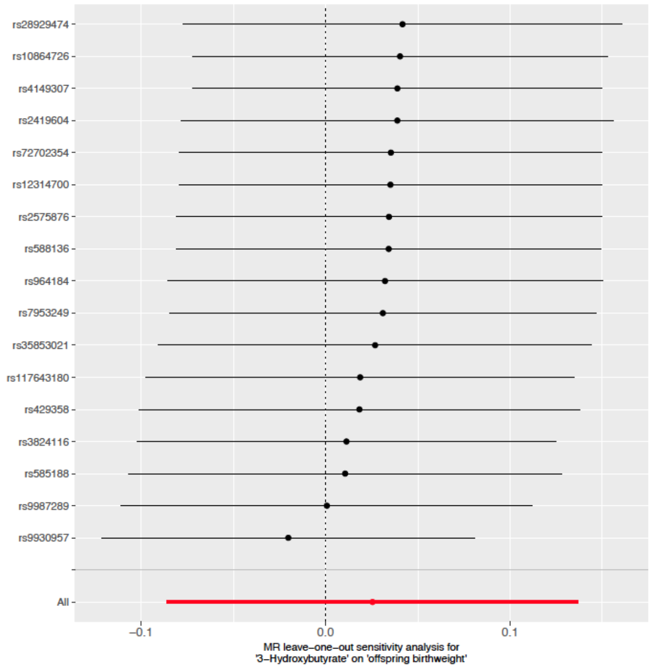
rs28929474
rs10864726
rs4149307
rs2419604
rs72702354
rs12314700
rs2575876
rs588136
rs964184
rs7953249
rs35853021
rs117643180
rs429358
rs3824116
rs585188
rs9987289
rs9930957
All
−0.1
0.0
0.1
MR leave−one−out sensitivity analysis for
'3−Hydroxybutyrate' on 'offspring birthweight'

(B)

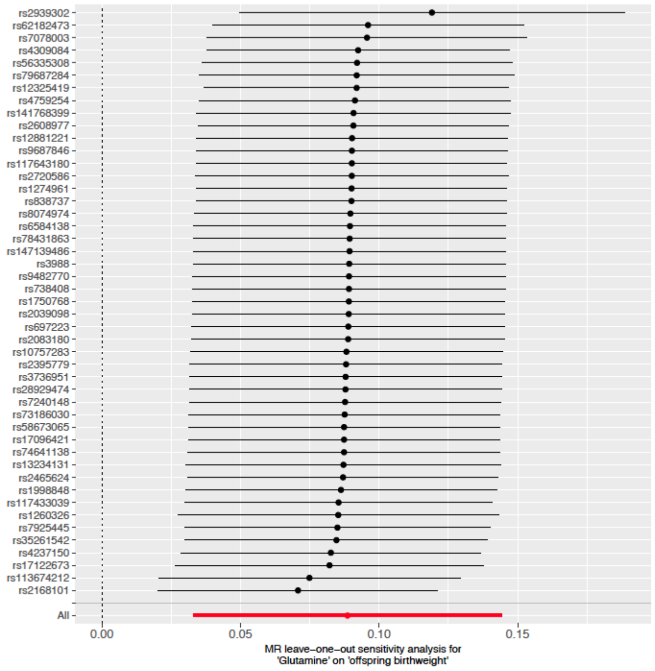
rs2939302
rs62182473
rs7078003
rs4309084
rs56335308
rs79687284
rs12325419
rs4759254
rs141768399
rs2608977
rs12881221
rs9687846
rs117643180
rs2720586
rs1274961
rs838737
rs8074974
rs6584138
rs78431863
rs147139486
rs3988
rs9482770
rs738408
rs1750768
rs2039098
rs697223
rs2083180
rs10757283
rs2395779
rs3736951
rs28929474
rs7240148
rs73186030
rs58673065
rs17096421
rs74641138
rs13234131
rs2465624
rs1998848
rs117433039
rs1260326
rs7925445
rs35261542
rs4237150
rs17122673
rs113674212
rs2168101
All
0.00
0.05
0.10
0.15
MR leave−one−out sensitivity analysis for
'Glutamine' on 'offspring birthweight'

(C)

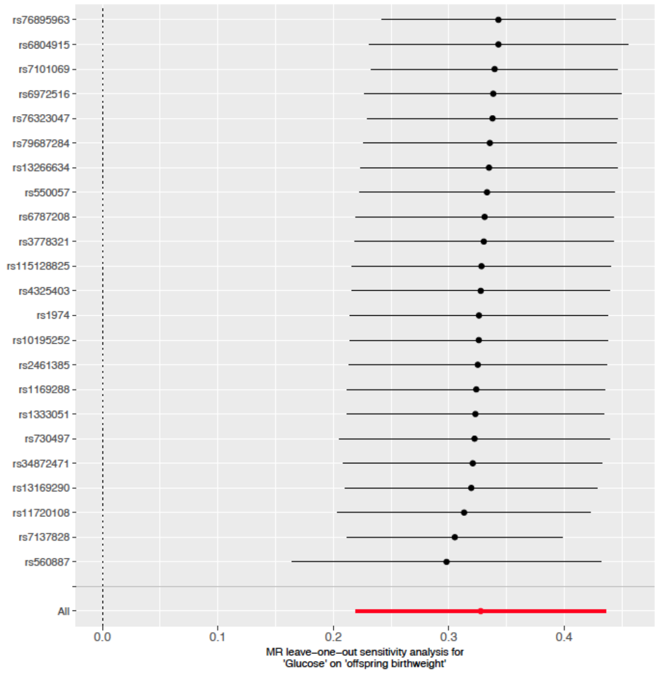
rs76895963
rs6804915
rs7101069
rs6972516
rs76323047
rs79687284
rs13266634
rs550057
rs6787208
rs3778321
rs115128825
rs4325403
rs1974
rs10195252
rs2461385
rs1169288
rs1333051
rs730497
rs34872471
rs13169290
rs11720108
rs7137828
rs560887
All
0.0
0.1
0.2
0.3
0.4
MR leave–one–out sensitivity analysis for
'Glucose' on 'offspring birthweight'

(D)

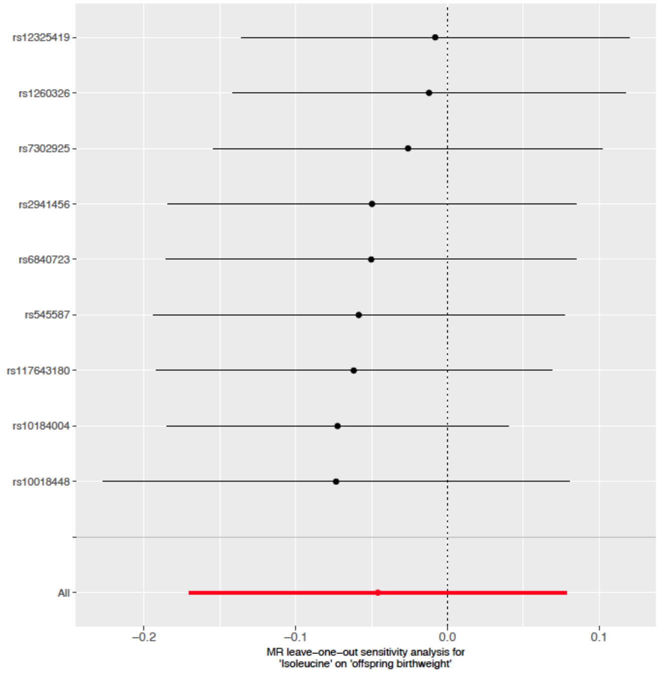
rs12325419
rs1260326
rs7302925
rs2941456
rs6840723
rs545587
rs117643180
rs10184004
rs10018448
All
−0.2
−0.1
0.0
0.1
MR leave–one–out sensitivity analysis for
'Isoleucine' on 'offspring birthweight'

(E)

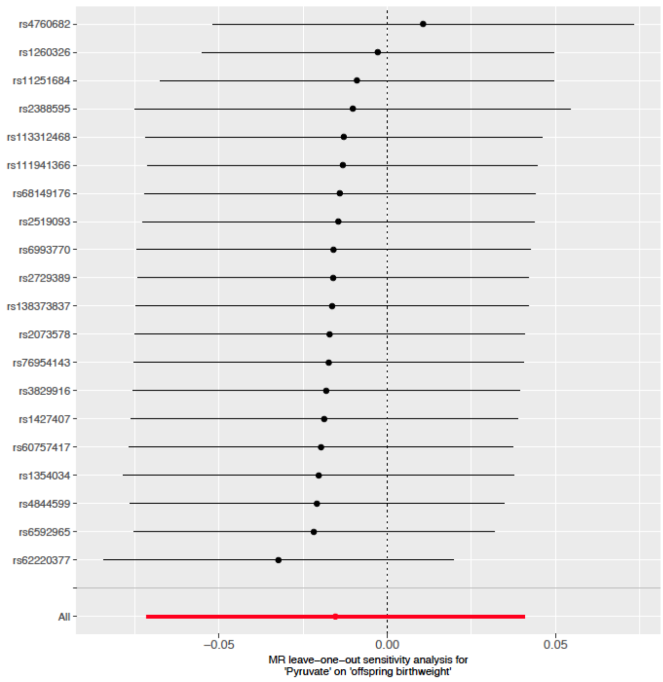

(F)

Supplementary Figures S1 (A)-(F): Plots of the leave-one-out sensitivity analysis of the IVW MR analysis excluding the specified SNP; a) alanine, b) 3-hydroxybutyrate, c) glutamine, d) glucose, e) isoleucine, f) pyruvate.

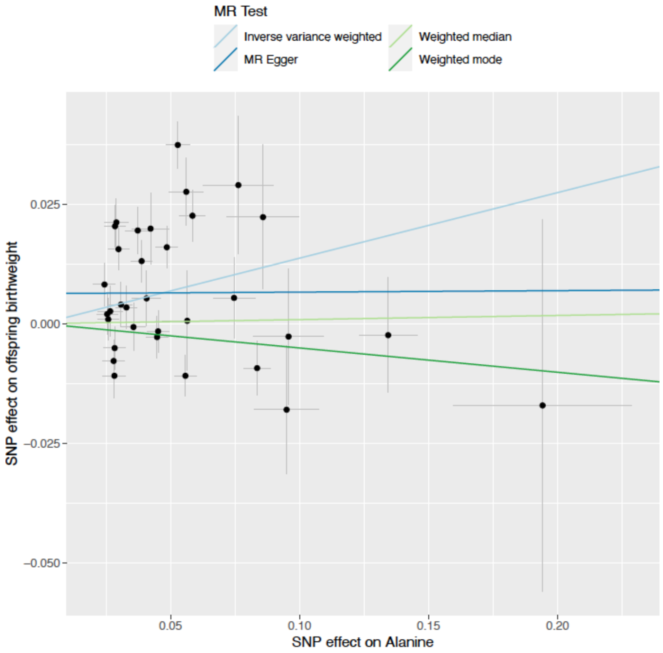

(A)

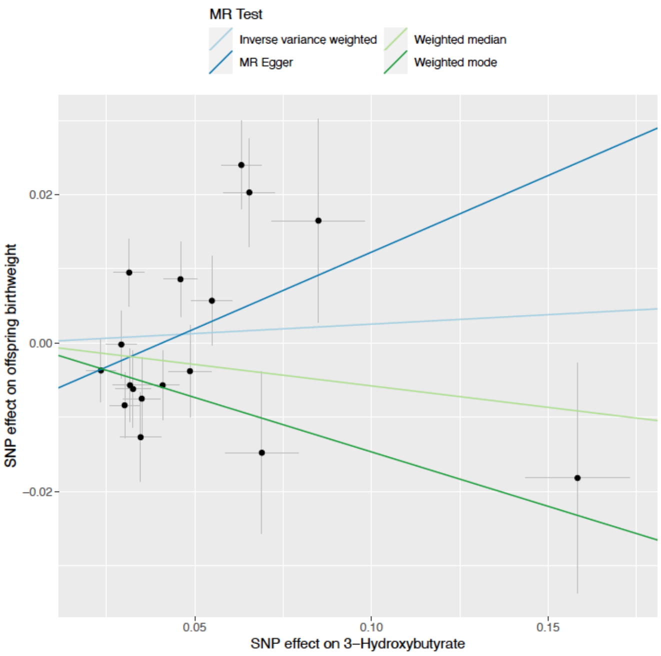
MR Test
Inverse variance weighted
MR Egger
Weighted median
Weighted mode
0.02
0.00
−0.02
SNP effect on offspring birthweight
0.05
0.10
0.15
SNP effect on 3−Hydroxybutyrate

(B)

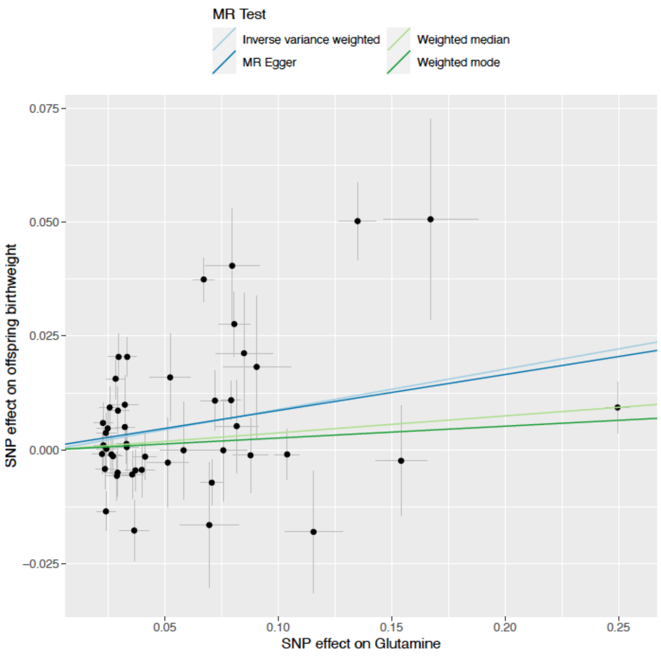
MR Test
Inverse variance weighted
MR Egger
Weighted median
Weighted mode
0.075
0.050
0.025
0.000
−0.025
SNP effect on offspring birthweight
0.05
0.10
0.15
0.20
0.25
SNP effect on Glutamine

(C)

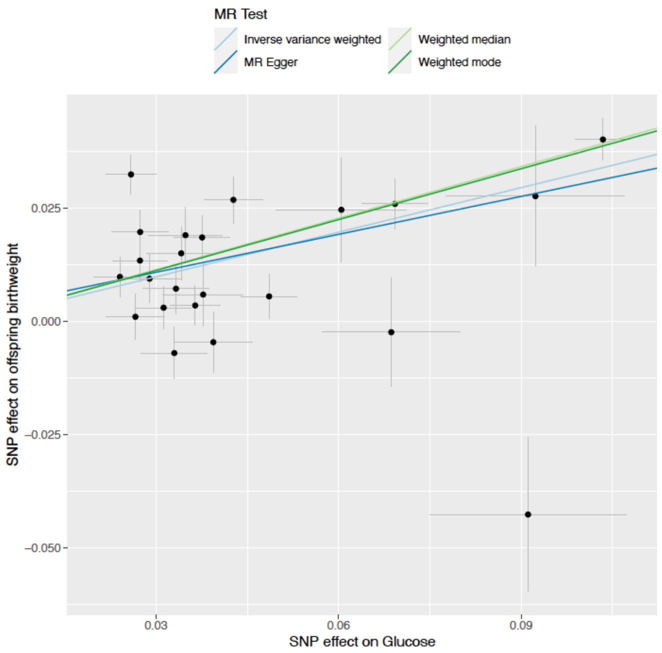
MR Test
Inverse variance weighted
MR Egger
Weighted median
Weighted mode
SNP effect on offspring birthweight
0.025
0.000
−0.025
−0.050
0.03
0.06
0.09
SNP effect on Glucose

(D)

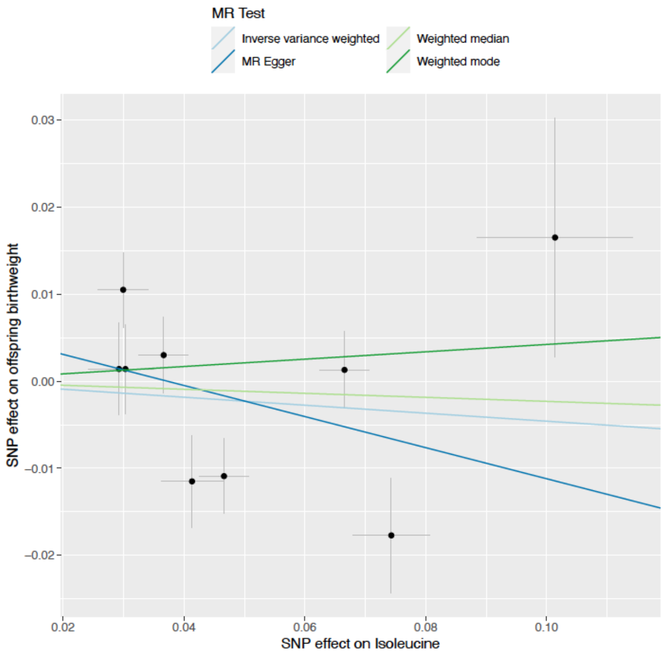
MR Test
Inverse variance weighted
MR Egger
Weighted median
Weighted mode
SNP effect on offspring birthweight
0.03
0.02
0.01
0.00
−0.01
−0.02
0.02
0.04
0.06
0.08
0.10
SNP effect on Isoleucine

(E)

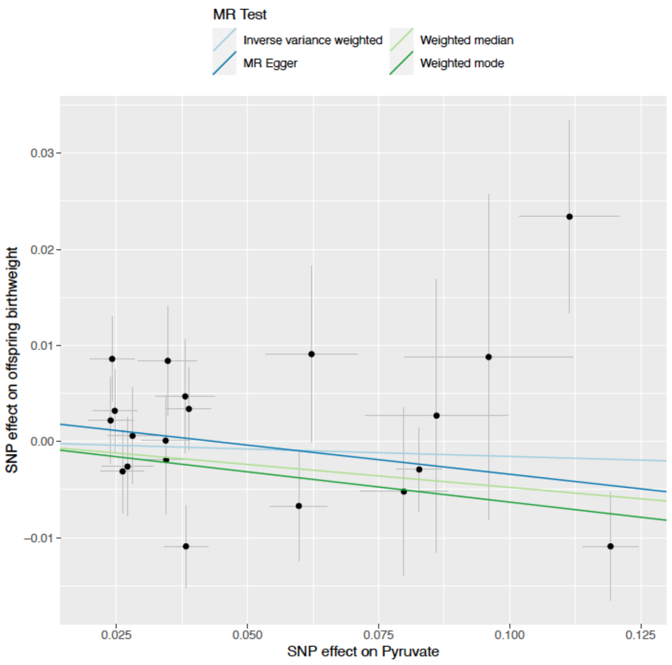

(F)

Supplementary Figures S2 (A)-(F): Scatter plots of UVMR analyses using IVW, MR-Egger, weighted median and weighted mode; a) alanine, b) 3-hydroxybutyrate, c) glutamine, d) glucose, e) isoleucine, f) pyruvate.

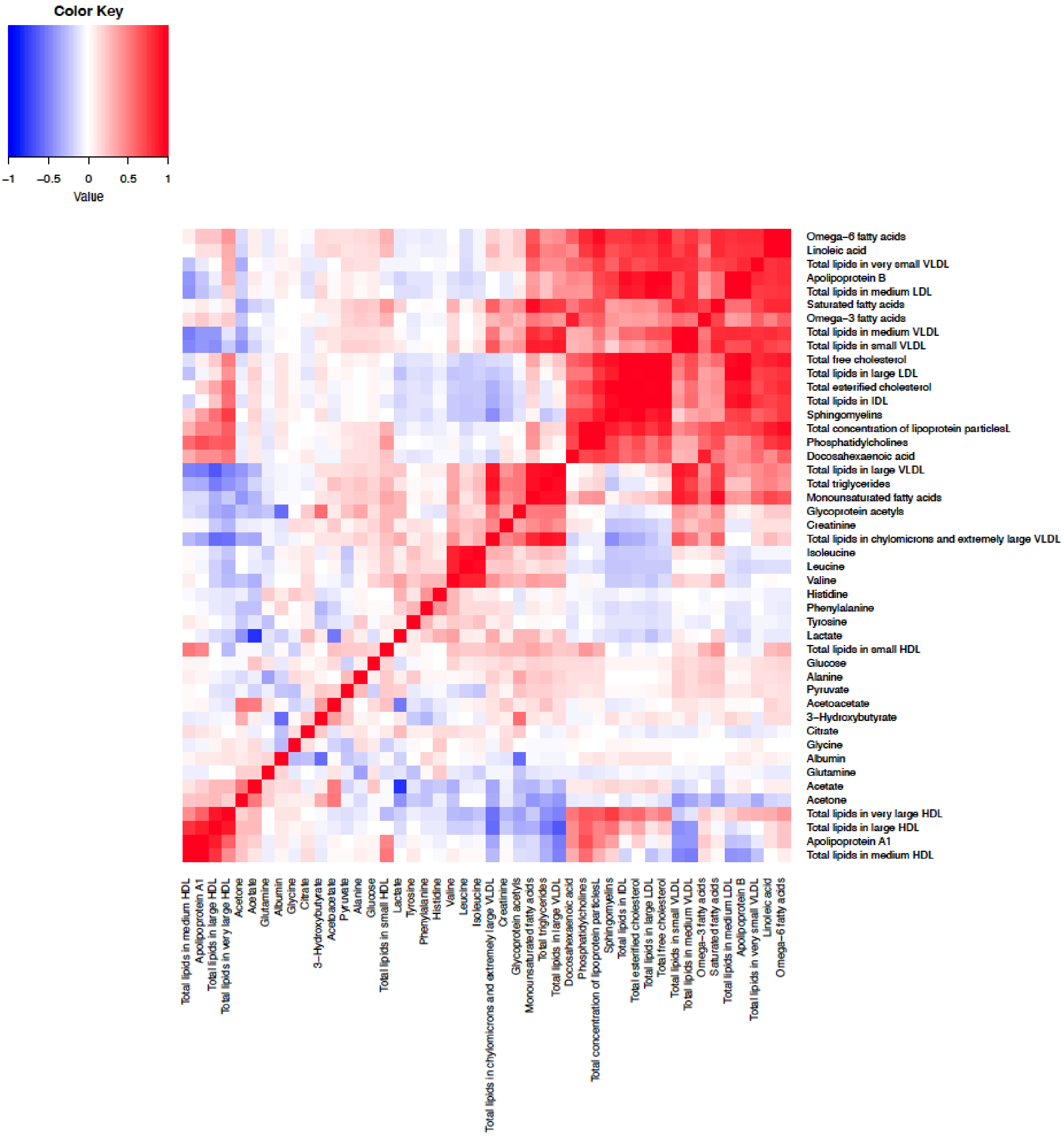

Supplementary Figure S3. Genetic correlation between metabolic measures based on n=114 harmonised SNPs within the Kettunen selected SNPs.

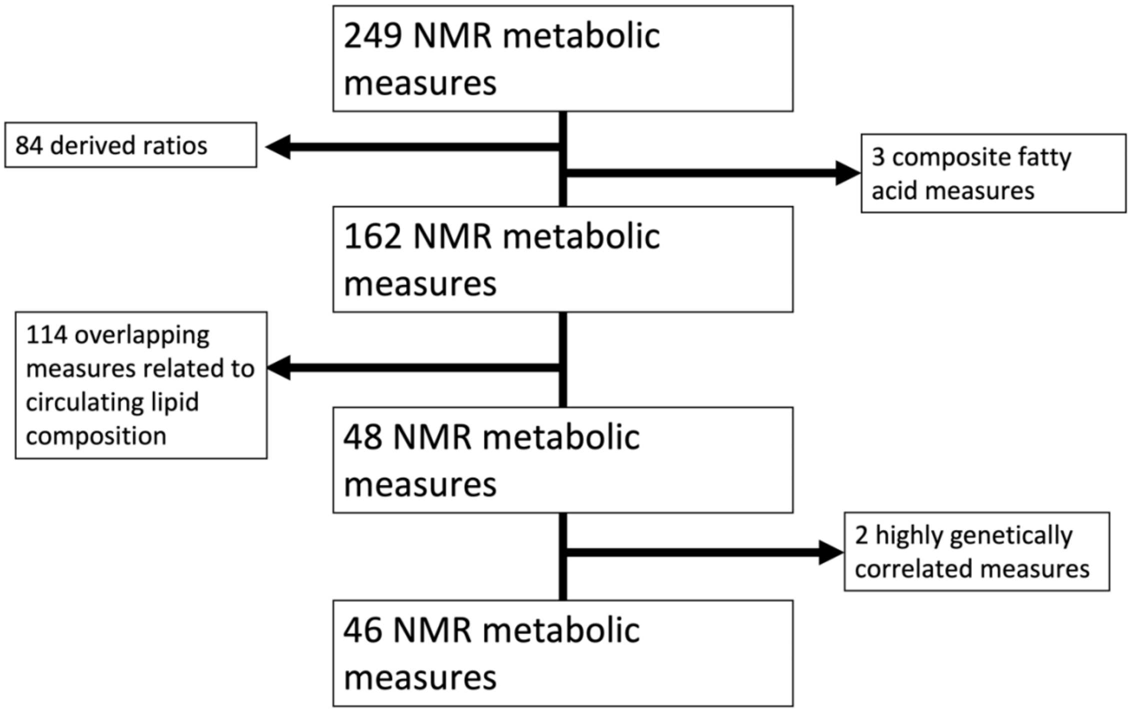

Supplementary Figure S4. Exclusions made to establish the list of metabolites.
